# Living without insulin: the role of leptin signaling in the hypothalamus

**DOI:** 10.3389/fnins.2015.00108

**Published:** 2015-03-27

**Authors:** Teppei Fujikawa, Roberto Coppari

**Affiliations:** ^1^Division of Hypothalamic Research, Department of Internal Medicine, The University of Texas Southwestern Medical CenterDallas, TX, USA; ^2^Department of Cellular Physiology and Metabolism, University of GenevaGeneva, Switzerland

**Keywords:** leptin, leptin receptors, insulin, diabetes mellitus, diabetes mellitus type 1, diabetes mellitus type 2, central nervous system

## Abstract

Since its discovery in 1922, insulin has been thought to be required for normal metabolic homeostasis and survival. However, this view would need to be revised as recent results from different laboratories have convincingly indicated that life without insulin is possible in rodent models. These data indicate that particular neuronal circuitries, which include hypothalamic leptin-responsive neurons, are empowered with the capability of permitting life in complete absence of insulin. Here, we review the neuronal and peripheral mechanisms by which leptin signaling in the central nervous system (CNS) regulates glucose metabolism in an insulin-independent manner.

## Introduction

The concept that “living without insulin is possible” may sound senseless as insulin signaling has long been thought to be essential for normal development and coordinated metabolic processes (e.g., glucose and lipid handling) in several species (e.g., from worms to humans) (Kenyon, 2010). However, recent findings indicate that insulin is dispensable for normal glucose and lipid metabolism as leptin monotherapy rescues glucose and lipid metabolic aberrancies and lethality caused by insulin deficiency, at least, in adult rodents (Yu et al., 2008; Fujikawa et al., 2010, 2013; Wang et al., 2010; Lee et al., 2012a). These results are interesting as we may be able to exploit the mechanisms underlying leptin's anti-diabetic action to develop better strategies against diabetes (Fujikawa et al., 2010, 2013). Since the very beginning, leptin has been shown to regulate glucose metabolism independently to its food-intake-suppressing action (Pelleymounter et al., 1995). Nevertheless, we suspect that no one could have imagined that leptin can regulate glucose metabolism in an insulin-independent manner at that time. Shortly after, researchers began realizing that leptin's effects on glucose metabolism might be separated into an insulin-dependent and -independent manner (Haque et al., 1999; Shimomura et al., 1999; Yaspelkis et al., 1999; Hidaka et al., 2002). Also, several scientific evidences indicate that the majority (if not all) of the glucose-lowering effect of leptin arises from its action on hypothalamic neurons (Coppari et al., 2005; Huo et al., 2009; Berglund et al., 2012). Yet, the concrete evidence supporting that leptin signaling in the CNS can regulate glucose homeostasis independently of insulin emerged only very recently.

Nobody seriously examined the possibility that “living without insulin is possible” until Dr. Roger Unger and his colleagues at University of Texas Southwestern Medical School in Dallas (USA) set out to tackle this possibility. They found that systemic overexpression of leptin ameliorates hyperglycemia in a near-complete insulin-deficient mouse model (Yu et al., 2008). Next, we revealed that the CNS is key to regulate glucose homeostasis in response to leptin in a similar insulin-deficient mouse model (Fujikawa et al., 2010). Furthermore, we demonstrated that intracerebroventricular (i.c.v.) leptin administration ameliorates hyperglycemia in complete insulin-deficient mice bearing a genetically-engineered gene that allows ablation of almost 100% of pancreatic β-cells; a degree of β-cells deficiency that is not achievable with any other animal model to date (Fujikawa et al., 2013).

Thus, almost 100 years after insulin was discovered, we now realize that mammalian organisms are equipped with neuro-circuities that can be exploited to restore normal glucose metabolism in the insulin-deficient setting. Yet, the exact components of this remarkable circuit have yet to be fully identified. One important issue need however to be considered. Because the amount of leptin needed to achieve virtually normal glucose and lipid metabolism in the absence of insulin is supraphysiological (Wang et al., 2010), it is very likely that these CNS circuitries have little physiological meaning. Nevertheless, identification of the underling mechanisms represents a unique opportunity to unmask molecular components which can then eventually become effective targets for better anti-diabetic drugs. Firstly, leptin therapy itself is unlikely to be effective in improving diabetes in humans because of the known leptin-resistant characteristic of these subjects (Myers et al., 2012). Secondly, because insulin is not required it is plausible that harnessing these molecular mechanisms could represent an ideal avenue to improve both types of diabetes; the insulin-deficient type 1 (T1DM) and the insulin-resistant type 2 diabetes mellitus (T2DM). Obviously, major efforts aimed at identifying these new putative anti-diabetes target(s) are currently underway.

In this review, we discuss the mechanism by which leptin signaling in the CNS regulates glucose metabolism in the absence of insulin.

## Leptin regulates glucose metabolism in insulin-dependent and -independent manner

In 1994, leptin was found by Friedman and colleagues who used positional cloning method to identify the mutation causing massive obesity in *ob/ob* mice (Zhang et al., 1994). A year later, leptin receptors (LEPRs) were also identified (Tartaglia et al., 1995; Chen et al., 1996). Leptin is expressed primary in adipose tissues (Zhang et al., 1994) and LEPRs are intensively expressed in the CNS (Gautron and Elmquist, 2011), although LEPRs expression is found in peripheral tissues as well (Margetic et al., 2002; Muoio and Lynis Dohm, 2002). In 1995, it was already known that leptin administration at a dose that does not affect body weight and food intake can restore normoglycemia in otherwise hyperglycemic *ob/ob* mice (Pelleymounter et al., 1995), suggesting indeed an important and direct role of leptin in coordinating glucose metabolism. Originally, leptin action on glucose metabolism appeared to be mediated by its insulin sensitizing effects (Schwartz et al., 1996; Barzilai et al., 1997). For example, systemic leptin administration dramatically reduces glucose levels along with insulin levels in otherwise hyperglycemic and hyperinsulinemic *ob/ob* mice (Schwartz et al., 1996). Leptin administration increases peripheral glucose uptake during insulin clamp (Barzilai et al., 1997), which support the notion that leptin is an insulin-sensitizer cue.

Kamohara and colleagues firstly pointed out the possibility that leptin's action on glucose metabolism may be classified into in an insulin-dependent and -independent manner (Kamohara et al., 1997). They found that either systemic (intravenous) or i.c.v. leptin administration increases glucose uptake in skeletal muscle and brown adipose tissue (BAT). Although their report did not provide concrete evidence, authors speculated that the glucoregulatory action of leptin might be executed independently of insulin (Kamohara et al., 1997). Chinookoswong and colleagues demonstrated that systemic leptin administration restores normoglycemia in otherwise hyperglycemic rats with low circulating insulin levels (~70 pg/mL) (Chinookoswong et al., 1999). Hidaka and colleagues found that leptin injection into the third cerebral ventricle also improves hyperglycemia in a similar animal model (Hidaka et al., 2002). Hence, collectively these results suggested that leptin exerts glycemia-lowering action by sensitizing metabolically relevant tissues to insulin but also in an insulin-independent manner.

There is no doubt that the aforementioned studies represent milestones in the process of recognizing the ability of leptin signaling in the CNS to ameliorate hyperglycemia in animal models of hypoinsulinemia. However, because insulin was still present (albeit at low levels) these studies were not able to address the crucial question whether insulin is required for the anti-diabetic action of leptin (Shimomura et al., 1999). In fact, it is possible that leptin signaling in the brain can hypersensitize peripheral tissues to insulin in a way that even low level of insulin is sufficient to maintain normal glucose levels in the blood in these otherwise diabetic animals.

In the following years researchers continued to use this hypoinsulinemic rodent model, but Unger and colleagues re-highlighted the action of leptin on glucose metabolism by generating and testing leptin's anti-diabetic action in near-complete insulin-deficient mice. They used a high-dose streptozotocin (STZ)-based approach to ablate the vast majority of pancreatic β-cells. STZ is a chemical that enters cells expressing glucose transporter 2 (GLUT2) and that destroys DNA in those cells (Lenzen, 2008). Using their pharmacological β-cells ablation model, Unger and colleagues generated near complete insulin-deficient mice (in this model insulin level is below 5 pg/mL, which is the threshold of detection of the most sensitive ELISA method used to quantify insulin), and demonstrated that systemic overexpression of leptin alleviates hyperglycemia in these mice (Yu et al., 2008). Moreover, their preclinical study shows that continuous systemic leptin administration ameliorates hyperglycemia and that it can reduce dosage of insulin for anti-diabetic treatment, suggesting that leptin may be applicable in the clinical setting as an adjuvant to insulin therapy (Wang et al., 2010).

The action of leptin on glucose metabolism in near complete insulin-deficient mice is mediated by the CNS (Fujikawa et al., 2010). In fact, i.c.v. leptin treatment recapitulates virtually all the metabolic improvements caused by systemic leptin administration in near complete insulin-deficient mice (Fujikawa et al., 2010). Combined with earlier work, these results then suggest that the CNS is key to leptin's action on glucose metabolism in an insulin-independent manner, as well as in the context of insulin sufficiency (Pelleymounter et al., 1995; Kamohara et al., 1997). Although the pharmacological approach used by Unger and colleagues can bring about virtually undetectable circulating insulin level, it cannot completely eliminate insulin-positive cells in the pancreas (Yu et al., 2008; Fujikawa et al., 2010). Thus, these studies were not able to rule in or out the possibility that the tiny amount of pancreatic insulin somehow underlies the anti-diabetic action of i.c.v. leptin administration. To address this issue we used a genetic tool generated by Thorel et al. They developed a mouse model bearing diphtheria toxin (DT) receptor gene driven by the rat-insulin promoter (RIP-DTR mice) (Thorel et al., 2010). DTR is not expressed in experimental mice (Saito et al., 2001), thus DT injections only affect and eradicate the cells expressing DTR. Following DT injections, RIP-DTR mice show ablation of almost 100% of pancreatic β-cells (Thorel et al., 2010; Fujikawa et al., 2013). DT-treated RIP-DTR mice exhibit no circulating insulin, and their pancreatic insulin is barely detectable; this is a degree of β-cell ablation far superior to the one achieved by STZ injections (Thorel et al., 2010). Of note, i.c.v. leptin treatment reverses hyperglycemia and permits survival of DT-treated RIP-DTR mice (Fujikawa et al., 2013).

**Figure 1:**
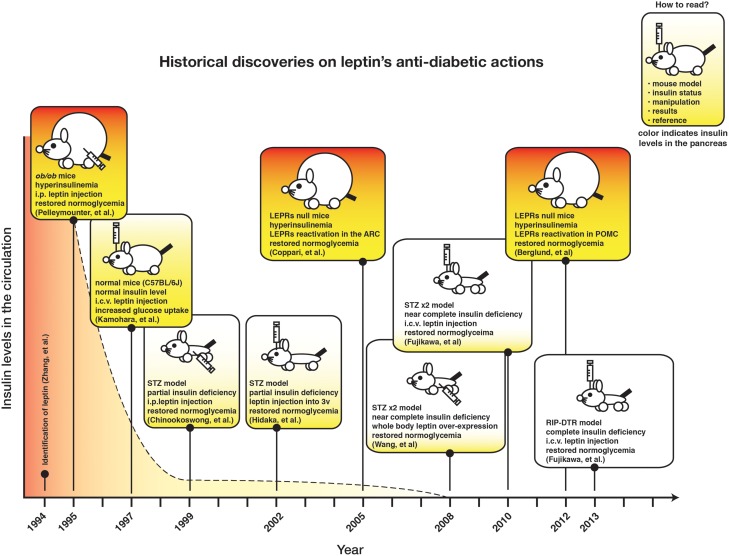
**The timeline of historical findings of leptin's anti-diabetic actions**.

Figure 1 depicts the chronicle of crucial discoveries on leptin's action on glucose metabolism. Although recurrent observations demonstrate that leptin signaling in the CNS can regulate glucose metabolism in insulin-deficient rodents, the neuronal mechanism underlying this action has just started to be understood.

## Diversity of neuronal networks underlying leptin's action on glucose metabolism

The discovery of leptin opened up the door of the genetic era in the field investigating the mechanism by which the CNS regulates metabolic homeostasis. Since then, researchers have focused on understanding the molecular mechanism by which the CNS regulates metabolism. LEPRs are broadly expressed in the brain including in the hypothalamus and brain stem which are well known to regulate metabolism. It is safe to say that the vast majority of the physiological function of leptin is mediated by LEPRs in the CNS, although LEPRs are expressed in peripheral tissues as well (Margetic et al., 2002; Muoio and Lynis Dohm, 2002). A large number of studies have unraveled the mechanism underlying leptin's action on food intake and body weight regulation (Gautron and Elmquist, 2011; Coppari and Bjorbaek, 2012), however the precise mechanism underlying leptin's action on glucose metabolism in the context of insulin-intact and -deficient models are still largely unknown.

Within the CNS, the hypothalamus is a key region for the regulation of glucose metabolism by leptin. Minokoshi et al. showed that microinjection of leptin into the ventromedial hypothalamic nuclei (VMH) of rats recapitulates the effect of i.c.v. leptin administration on glucose uptake in peripheral tissues (Minokoshi et al., 1999). In 2005, we reported that direct action of leptin on LEPRs in the hypothalamic arcuate nucleus (ARC), which is located in the ventral region of the hypothalamus, is sufficient to restore normoglycemia in otherwise hyperglycemic LEPRs null mice (Coppari et al., 2005). Huo et al. demonstrated that over-expression of LEPR-b, which is a biological isoform of LEPRs, in all proopiomelanocortin (POMC) neurons is sufficient to achieve similar glucose improvements in otherwise LEPRs null mice (Huo et al., 2009). Given that the leptin-responsive neurons appear to be ~30% of POMC neurons in the ARC (Williams et al., 2010) and the over-expression of LEPR-b under cytomegalovirus promoter forces LEPRs to be expressed at super-physiological levels in all POMC neurons, the physiological role of leptin-responsive POMC neurons was still unclear at that time. Later, Berglund et al. found that reactivation of endogenous LEPRs only in POMC neurons is sufficient to restore normoglycemia in otherwise hyperglycemic LEPRs null mice; this glucose effect was seen, despite no changes in body weight and food intake (Berglund et al., 2012). Reactivation of LEPRs only in POMC neurons restores insulin sensitivity in the liver and suppresses hyperglucagonemia in mice otherwise displaying severe hepatic insulin resistance and elevated level of circulating glucagon (Berglund et al., 2012). Other studies have shown the importance of POMC neurons on glucose metabolism (Kievit et al., 2006; Parton et al., 2007; Hill et al., 2009, 2010).

Similarly to other biological systems, LEPRs network in the CNS likely is a redundant system. This seems to be particularly true for its effect on body weight (Balthasar et al., 2004; Dhillon et al., 2006; van de Wall et al., 2008; Hill et al., 2010; Leinninger et al., 2011; Vong et al., 2011; Leshan et al., 2012; Dodd et al., 2014), and may be on glucose metabolism as well. Agouti-related peptide (AgRP) is expressed in the juxtaposed-neurons to POMC neurons in the ARC and 10-20% of AgRP neurons are leptin-responsive neurons (Mizuno and Mobbs, 1999; Wilson et al., 1999). Recently, Goncalves et al. found that over-expression of LEPR-b in all AgRP neurons of rescues hyperglycemia in otherwise LEPRs null mice and deletion of LEPRs only in AgRP neurons diminishes leptin's glucose lowering effects in the context of T2DM (Goncalves et al., 2014). Again, only 10–20% of AgRP neurons express LEPR-b (Wilson et al., 1999), thus the physiological function of endogenous LEPRs in AgRP neurons is still undetermined. Nonetheless, LEPRs in AgRP neurons may play a key role in the regulation of glucose metabolism as well as POMC neurons do. Further studies will be needed to firmly establish the role of LEPRs in AgRP neurons on glucose homeostasis in the context of T1DM and T2DM.

The results from studies performed in the context of insulin-intact rodents indicate that distinct chemically identified neurons, such as POMC neurons, are important for the regulation of glucose homeostasis. One can imagine that the same neuronal LEPRs system also mediate anti-diabetic actions of leptin in the context of complete-insulin deficiency. Surprisingly, our recent study indicates that this is not the case. LEPRs in POMC neurons are sufficient to maintain glucose levels, but not required for normal glucose homeostasis in the context of insulin-intact model. In fact, reactivation of LEPRs only in POMC neurons is sufficient to ameliorate hyperglycemia in otherwise diabetic LEPRs null mice (Berglund et al., 2012), while deletion of LEPRs only in POMC neurons has no effect on blood glucose levels (Balthasar et al., 2004). Contrary to insulin-intact models, LEPRs in POMC neurons are not sufficient to restore normoglycemia, but are partially required for leptin's anti-diabetic actions in the context of T1DM (Fujikawa et al., 2013). Reactivation of LEPRs only in POMC neurons is not sufficient for achieving the anti-T1DM and lifesaving action of leptin administration, and deletion of LEPRs only in POMC neurons slightly hampers these actions of leptin (Fujikawa et al., 2013). Notably, despite the importance of LEPRs in the VMH on the regulation of glucose uptake in peripheral tissues (Minokoshi et al., 1999), LEPRs in VMH appear to be not essential for leptin's anti-T1DM and -T2DM actions. Over-expression of LEPRs in streroidgenic factor-1 (SF-1) neurons which are located exclusively in the VMH does not affect glucose levels in otherwise LEPRs null mice, and deletion of LEPRs in SF-1 neurons has no impact on leptin's anti-diabetic actions in the context of insulin-intact models (Goncalves et al., 2014). Deletion of LEPRs only in SF-1 neurons either does not affect leptin's anti-diabetic actions in insulin-deficient models (Fujikawa et al., 2013; Meek et al., 2013). Collectively, the aforementioned studies suggest that an array of LEPRs-expressing neurons is required for leptin's anti-diabetic actions in the absence of insulin while a single chemically-identified neuronal group is sufficient for mediating the anti-diabetic action of leptin in the presence of insulin (Huo et al., 2009; Berglund et al., 2012; Goncalves et al., 2014).

**Figure 2:**
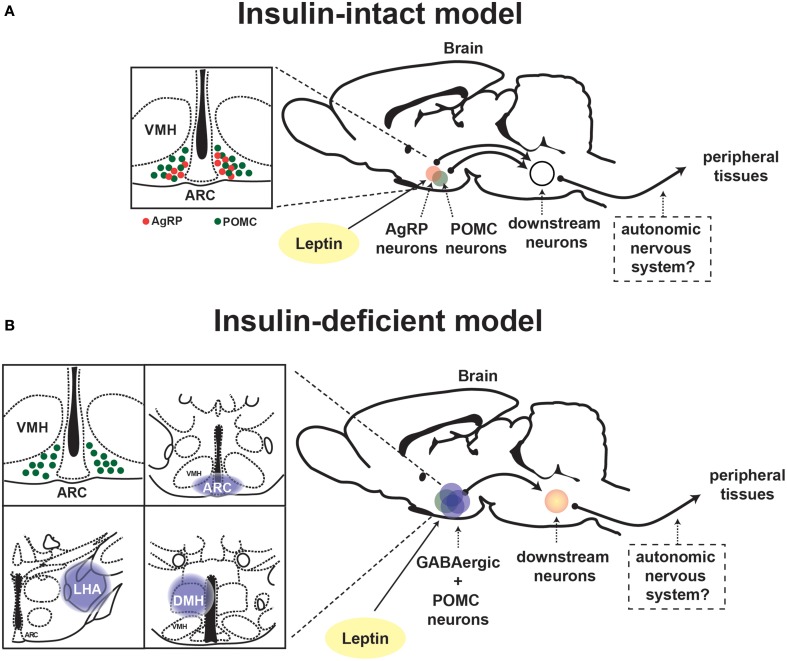
**Proposed neuronal pathway underlying leptin's anti-diabetic actions in the presence and absence of insulin**. Schematic depiction of neuronal circuit underlying leptin's anti-diabetic actions in **(A)** insulin-intact and **(B)** -deficient model. ARC, hypothalamic arcuate nucleus; DMH, dorsomedial nucleus; VMH, ventromedial hypothalamic nucleus; AgRP, Agouti-related peptide; POMC, proopiomelanocortin.

In general, neurons are classified into either glutamatergic excitatory or γ-aminobutyric (GABA)-ergic inhibitory neurons, although recent study suggests that some particular neurons can produce and release both glutamate and GABA as neurotransmitters (Root et al., 2014). Vong et al. investigated the fundamental query whether LEPRs in glutamatergic or GABAergic neurons are important for leptin's actions on physiological function. To this end, they generated mice expressing Cre-recombinase only in glutamatergic or GABAergic neurons which enable to manipulate LEPRs only in those neurons (Vong et al., 2011). Deletion of LEPRs only in GABAergic neurons leads to massive obesity, hyperphagia, hyperglycemia and other metabolic defects, which are of similar (yet not identical) magnitude as the ones seen in whole-body LEPR null mice (Vong et al., 2011). Contrary to deletion of LEPRs in GABAergic neurons, that only in glutamatergic neurons causes a slight body weight gain (Vong et al., 2011). The study provides the evidence that a distributed rather than a single neuronal group of LEPRs-expressing neurons plays a crucial role in leptin's action on energy balance. This notion is also applied to the mechanism underlying leptin's anti-diabetic actions in the absence of insulin. Reactivation of LEPRs only in GABAergic neurons is sufficient to mediate anti-diabetic actions of the hormone in the absence of insulin, although the survival rate is not fully restored (Fujikawa et al., 2013). Leptin administration into the brain of mice expressing LEPRs only in both GABAergic and POMC neurons completely restores normoglycemia in otherwise hyperglycemic mice and permit them to survive at similar degrees as the ones seen in LEPRs-intact controls (Fujikawa et al., 2013). These results indicate that leptin's anti-diabetic actions in the absence of insulin require a broad neuronal network. Figure 2 depicts the summary of leptin's diabetic actions in the presence and absence of insulin.

Within GABAergic neurons, the leptin-responsive GABAergic neurons are restrictedly located in the hypothalamus, in particular the ARC, dorsomedial hypothalamic nucleus (DMH), and lateral hypothalamic area (LHA) (Vianna and Coppari, 2011; Fujikawa et al., 2013). In the ARC, two neuronal populations are known as GABAergic neurons. AgRP neurons are almost 100% GABAergic in nature (Vong et al., 2011), and recently RIP-Cre neurons are identified as ARC GABAergic neurons as well (Kong et al., 2012). As described above, LEPRs in AgRP neurons may have a potential to mediate leptin's anti-diabetic actions in the absence of insulin (Goncalves et al., 2014). RIP-Cre expressing cells are broadly positioned in the CNS and pancreatic β-cells (Song et al., 2010; Wicksteed et al., 2010). RIP-Cre neurons are defined as neurons expressing Cre under rat-insulin promoter (*ins2*), in particular using a mouse strain *Tg(Ins2-cre)^25Mgn^* (Jackson laboratory, stock #003573) or *Tg(Ins2-Cre)^1Herr^* (Kong et al., 2012; Rother et al., 2012). Thus, we here describe RIP-Cre expressing cells in the CNS as RIP^25Mgn^-Cre or RIP^1Herr^-Cre neurons.

RIP^1Herr^-Cre neurons show far less Cre-activity in the CNS compared to RIP^25Mgn^-Cre neurons (Wicksteed et al., 2010; Rother et al., 2012), indicating that RIP-Cre neurons may not represent endogenous *ins2*-expressing cells in the CNS. Nonetheless, RIP^25Mgn^-Cre neurons in the ARC are distinguished from AgRP and POMC neurons (Kong et al., 2012), and are GABAergic in nature (Kong et al., 2012). Activation of RIP^25Mgn^-Cre neurons in the ARC causes thermogenesis (Kong et al., 2012) and it is well known that thermogenesis accompanies with increases in glucose uptake in peripheral tissues (Cypess et al., 2009; Virtanen et al., 2009; van Marken Lichtenbelt et al., 2009). Moreover, some of RIP^25Mgn^-Cre neurons in the ARC are leptin-responsive neurons (Kong et al., 2012). Thus, it is virtually possible that the leptin-responsive RIP^25Mgn^-Cre neurons play a crucial role in mediating the leptin's anti-diabetic actions in the absence of insulin through the thermogenesis-induced glucose uptake. Contrary to RIP^25Mgn^-Cre neurons, it is not clear whether RIP^1Herr^-Cre neurons are leptin-responsive. Ablation of RIP^1Herr^-Cre neurons dramatically reduces food intake and body weight, but it does not affect glucose tolerance, indicating that RIP^1Herr^-Cre neurons may not regulate glucose homeostasis (Rother et al., 2012).

LEPRs-expressing neurons in the DMH are known to regulate thermogenesis through the sympathetic nervous system (SNS) (Enriori et al., 2011; Dodd et al., 2014; Rezai-Zadeh et al., 2014). It is not clear whether those DMH neurons are GABAergic neurons. Additionally, it is still unknown whether LEPRs-expressing neurons in the DMH regulate glucose homeostasis, but again it is possible that they play a crucial role in leptin's anti-diabetic actions via thermogenesis. The chemical identity of GABAergic leptin-responsive DMH neurons is still unknown; however, several gene candidates have been proposed to characterize this neuronal group (e.g., *prl, Gpr50, Pcsk5, Sulf1, 4930511J11Rik, Grp*, and *Rorb*) (Lee et al., 2012b; Rezai-Zadeh et al., 2014). The leptin-responsive neurons in the LHA are GABAergic neurons, and regulate food intake (Leinninger et al., 2009). The role of the leptin-responsive neurons in the LHA in the regulation of glucose homeostasis is still unclear; however, they unlikely mediate leptin's anti-diabetic actions (Minokoshi et al., 1999; Leinninger et al., 2009, 2011). Collectively, the leptin-responsive neurons in the ARC and DMH are strong candidates for mediating leptin's anti-diabetic actions in the absence of insulin. Further studies are required to unravel the role of these neurons in regulating glucose homeostasis in the presence and absence of insulin.

## Downstream neuronal and molecular pathways of leptin's anti-diabetic actions in the absence of insulin

The neuronal substrates that lay downstream to the first-order LEPRs-expressing neurons underlying leptin's anti-diabetic action are largely unknown. This is true in the context of insulin-resistant and -deficient diabetes. The paraventricular hypothalamus (PVH) is a great candidate for conveying information from leptin-responsive neurons to downstream sites aimed at regulating glucose metabolism. Various neurons in the ARC including POMC, AgRP, RIP^25Mgn^-Cre and RIP^1Herr^-Cre neurons project to the PVH (Schwartz et al., 2000; Kong et al., 2012; Rother et al., 2012). POMC neurons release α-melanocyte-stimulating hormones (α-MSH) (Mains and Eipper, 1976; Cone, 2006), and AgRP neurons release AgRP (Lu et al., 2003). These neurotransmitters have opposite effects on the same melanocortin receptors (MCRs), and one of MCRs isoform, melanocortin receptor 4 (MC4R), is highly expressed in the PVH (Balthasar et al., 2005). Additionally, the leptin-responsive neurons in the DMH strongly connect to the PVH (Elmquist et al., 1998). A microinjection of noradrenalin or serotonin agonist into the PVH and a microdialysis of thyroid hormone in the PVH increase glucose levels in the blood and those actions are likely mediated by the SNS-liver axis (Ionescu et al., 1989; Korte et al., 1991; Klieverik et al., 2009). Moreover, corticotrophin-releasing hormone (CRH or CRF) causes hyperglycemia *via* the hypothalamus-pituitary-adrenal (HPA)-axis (Jamieson et al., 2006). These notions clearly suggest that the PVH regulates glucose homeostasis.

**Figure 3:**
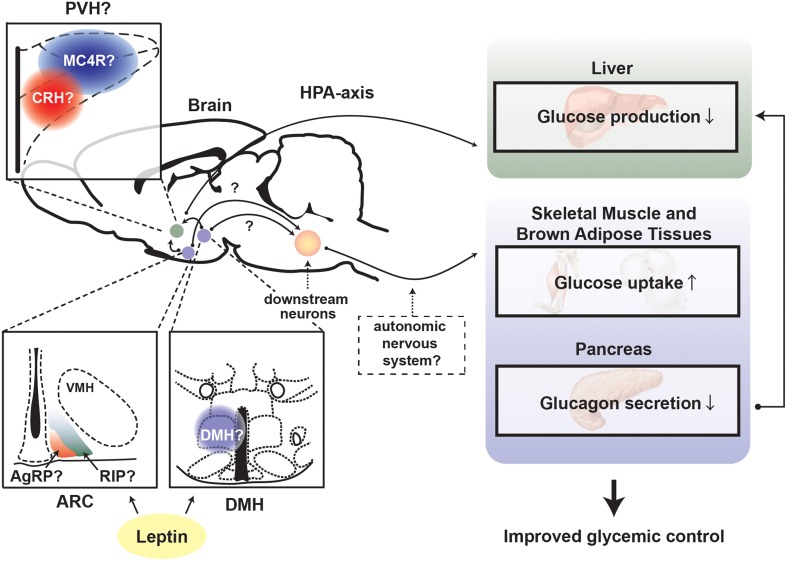
**Proposed mechanism underlying leptin's anti-diabetic actions in the absence of insulin**. Depiction of the proposed central pathways and peripheral tissues by which leptin signaling in the brain exerts anti-diabetic action in the absence of insulin. ARC, hypothalamic arcuate nucleus; DMH, dorsomedial nucleus; VMH, ventromedial hypothalamic nucleus; PVH, paraventricular hypothalamus; AgRP, Agouti-related peptide; RIP, rat insulin promoter; MC4R, melanocortin receptor 4; CRH, corticotrophin-releasing hormone; HPA-axis, hypothalamus-pituitary-adrenal (HPA)-axis.

Interestingly, a recent study suggests that the suppressing effect of leptin on overzealous HPA-axis activity caused by insulin deficiency is key for the anti-diabetic actions of leptin (Perry et al., 2014). Of note, whether leptin signaling in the CNS affects central and pituitary components of the HPA axis in complete insulin deficiency is still unknown. Nevertheless, MC4R is potentially a vital mediator for the suppressing effect of leptin on HPA-axis. MTII (agonist for MC3R/4R) administration into the brain stimulates HPA-axis and increases glucose levels, and either MC4R or CRH antagonist can diminish the glucose-elevating effect of MTII (Lu et al., 2003). Interestingly, MC4R is required for leptin's anti-diabetic actions in the context of T2DM (Goncalves et al., 2014). MC4R in the PVH likely regulates glucose homeostasis as reactivation of MC4R only in single-minded homolog 1 (SIM1) neurons, which represents the vast majority of PVH neurons, dramatically improves glucose homeostasis in otherwise MC4R null mice (Shah et al., 2014). Collectively, these studies indicate that leptin signaling in the brain regulates glucose homeostasis *via* the leptin-responsive neurons in the ARC and/or DMH and that these neurons signal to MC4R neurons in the PVH → CRH neurons → HPA-axis to suppress hepatic glucose production (Figure 3).

As mentioned above, the SNS may also be a crucial downstream component that facilitates leptin's anti-diabetic actions in the absence of insulin. Leptin administration into the brain enhances glucose uptake in the skeletal muscle and BAT, and resection of nerves to skeletal muscle completely blocks the action of leptin on glucose uptake (Kamohara et al., 1997), suggesting that leptin signaling in the brain regulates glucose uptake via the SNS. In the presence of insulin, b-adrenergic receptors are likely required for the action of leptin on glucose uptake (Haque et al., 1999), but those receptors are not necessary for leptin's anti-diabetic actions in the absence of insulin (Fujikawa et al., 2013). Injections of α-adrenergic receptors agonist can increase glucose uptake in the heart (Doenst and Taegtmeyer, 1999), indicating that these receptors may be involved in leptin's anti-diabetic actions in the absence of insulin.

The parasympathetic nervous system (PNS), which is another arm of the autonomic nervous system, may also be involved in the mechanism underlying leptin's anti-diabetic actions in the absence of insulin. Suppressing action of leptin on glucagon secretion has been suggested to be crucial for leptin's anti-diabetic actions (Yu et al., 2008; Wang et al., 2010; Unger and Cherrington, 2012). Both the SNS and PNS regulate secretion of pancreatic glucagon. For instance, stimulation of either the SNS or PNS increases glucagon secretion from pancreatic α-cells (Marliss et al., 1973; Kaneto et al., 1974; Bloom and Edwards, 1978). The PNS projections to the pancreas is made of cholinergic neurons and blocking muscarinic receptors by atropine reduces the basal levels of glucagon (Bloom et al., 1974). It is completely unknown whether leptin signaling in the brain can modulate the PNS; however, it may be possible that inhibitory action of leptin on glucagon secretion is mediated by the PNS. It is formally possible that centrally-delivered leptin leaks to the systemic circulation hence acting on pancreatic α-cells to directly inhibit glucagon secretion. However, our results indicate that pancreatic α-cells are devoid of LEPR-b in the presence and even in the absence of insulin (Fujikawa et al., 2013).

Intriguingly, recent studies have indicated that LEPRs in non-neuronal cells in the brain are important for mediating leptin's physiological function (Fuente-Martin et al., 2012; Balland et al., 2014; Kim et al., 2014). Astrocytes have a capability of sensing alternations in energy, iron and pH levels, and may be involved in coordinated responses aimed at maintaining these parameters within the normal range (Garcia-Caceres et al., 2013). Fuente-Martín et al. demonstrated that leptin can modify glutamate and glucose transporter expression levels as well as glutamate and glucose uptake in hypothalamic astrocyte (Fuente-Martin et al., 2012). Moreover, Kim et al. showed that deletion of LEPRs specifically in astrocyte using a glial fibrillary acidic protein (GFAP)-Cre mouse line attenuated anorexinergic effect of leptin and enhanced orexinergic effect of ghrelin (Kim et al., 2014). Tanycytes that are glia-like cells and line the third ventricle and median eminence (ME) have emerged as a key communicator between cerebrospinal fluid and hypothalamic neurons to regulate whole-body metabolism (Bolborea and Dale, 2013). LEPRs on tanycytes in the ME serve as transporter of leptin from blood to hypothalamic neurons, and high-fat diet can disrupt leptin signaling in these cells; an effect suggested to be contributing to leptin resistance seen in this context (Balland et al., 2014). Although none of aforementioned studies show that LEPRs in astrocytes or tanycytes are involved in the regulation of glucose metabolism, it is virtually possible that they can complement the actions of leptin on neurons on metabolic homeostasis. Future studies will be required to address this possibility.

## Targeted peripheral tissues for the leptin's anti-diabetic actions

Leptin signaling in the brain governs many peripheral tissues with the goal of maintaining glucose homeostasis. One of these tissues is likely the liver. Insulin deficiency accelerates gluconeogenesis and glycogenolysis, hence causing exaggerated hepatic glucose production. Overexpression of LEPRs in the ARC in LEPRs null rats restores normal hepatic glucose production (German et al., 2009). Moreover, re-expression of LEPRs only in POMC neurons is sufficient to restore normal hepatic glucose production in otherwise over-productive rodents, suggesting that leptin-responsive POMC neurons regulate hepatic glucose production; this effect is likely due to sensitization of insulin action on hepatocytes (Berglund et al., 2012).

The specific mechanism by which leptin signaling in the brain regulates hepatic glucose production is still unclear but there are two pathways that may be involved in mediating leptin's actions on the liver. One pathway is the PNS as hepatic branch vagatomy (resection of vagus nerve projecting to the liver and other visceral tissues) attenuates leptin's insulin-sensitizing action on the liver (German et al., 2009). Another pathway is the HPA-axis as described above. Leptin administration can suppress hyper-activated HPA axis brought on by STZ-induced insulin reduction, hence reduce corticosterone levels in the blood that otherwise would stimulate gluconeogenesis (Perry et al., 2014). Part of exaggerated hepatic glucose production likely results from hyperglucagonemia induced by insulin deficiency. Insulin suppresses glucagon secretion from pancreatic α-cells and glucagon is one of the most potent hormones to induce gluconeogenesis (Unger and Cherrington, 2012). Leptin administration into the brain completely suppresses hyperglucagonemia in the absence of insulin (Fujikawa et al., 2010, 2013).

Leptin administration into the brain enhances glucose uptake in skeletal muscle and BAT in the presence of insulin (Kamohara et al., 1997). Evidences suggest that part of these actions is likely mediated by the VMH (Minokoshi et al., 1999). However, again, neither re-expression/overexpression nor deletion of LEPRs only in SF-1 neurons, which represent the VMH, affects anti-diabetic actions of leptin both in the presence and absence of insulin (Fujikawa et al., 2013; Goncalves et al., 2014); thus the importance of LEPRs in the VMH and enhancement of glucose uptake in those tissues is still unclear. Interestingly, subcutaneous transplantation of embryonic BAT can ameliorate STZ-induced diabetes (Gunawardana and Piston, 2012), suggesting that BAT may play a key role in the regulation of glucose metabolism brought on by leptin in the absence of insulin.

## Remarks and perspective

The last two decades have witnessed tremendous efforts aimed at understanding the mechanisms underlying leptin's anti-diabetic action in the presence or absence of insulin. Results from these studies raised the possibility of designing completely new therapeutics for the treatment of diabetes and other metabolic diseases. These studies on leptin's anti-diabetic actions also provide support to the notion that “living without insulin is possible.”

Because the current knowledge represent a starting point, in the near future we will need to understand the following: (i) the biochemical identity of neuronal populations that are crucial for leptin's anti-diabetic actions in the absence of insulin; (ii) whether these effects are due to changes in neuronal membrane activities, and/or transcription, and/or neurotransmitter secretion (Williams et al., 2011). If alternations in neuronal activity are required for the actions of leptin, we may be able to achieve normal glucose homeostasis in the absence of insulin, without leptin, just by changing the biophysical properties of crucial neurons. If the actions require transcriptional changes in targeted neurons and we can identify those genes, modifications in their expression may restore normoglycemia in the absence of insulin. There is little doubt that by unraveling the mechanism underlying the life-saving action of leptin therapy we will pave the way for developing better anti-diabetic approaches.

### Conflict of interest statement

The authors declare that the research was conducted in the absence of any commercial or financial relationships that could be construed as a potential conflict of interest.
